# Endoscopic Visualization of a Jejuno‐Colonic Fistula in a Case of Monomorphic Epitheliotropic Intestinal T‐Cell Lymphoma

**DOI:** 10.1002/deo2.70366

**Published:** 2026-06-15

**Authors:** Kanami Ota, Yuichi Matsuno, Yoshiaki Taniguchi, Noriyuki Imazu, Shinichiro Kawatoko, Yutaro Ihara, Tomohiro Nagasue, Keisuke Kawasaki, Tomohiko Moriyama, Junji Umeno

**Affiliations:** ^1^ Department of Medicine and Clinical Science Graduate School of Medical Sciences Kyushu University Fukuoka Japan; ^2^ Department of Anatomic Pathology Graduate School of Medical Sciences Kyushu University Fukuoka Japan

**Keywords:** endoscopic findings, enteropathy‐associated T‐cell lymphoma, jejuno‐colonic fistula, monomorphic epitheliotropic intestinal T‐cell lymphoma, small intestinal lymphoma

## Abstract

Monomorphic epitheliotropic intestinal T‐cell lymphoma (MEITL) is a rare primary intestinal T‐cell lymphoma newly defined in the 2016 WHO classification, characterized by rapid progression and a poor prognosis. Fistula formation caused by intestinal lymphoma is extremely uncommon, and no previous reports have described MEITL presenting with a jejunocolic fistula. An 83‐year‐old woman presented with a 20‐kg weight loss and generalized fatigue over the preceding year. Laboratory tests revealed hypoalbuminemia and elevated inflammatory markers. Computed tomography demonstrated wall thickening of the jejunum and descending colon, suggesting possible continuity between the two segments. Colonoscopy revealed a circumferential ulcerative lesion at the splenic flexure, and proximal to this lesion, another lumen extending in a direction different from the colonic lumen was observed. Biopsy confirmed a diagnosis of MEITL. Positron emission tomography–computed tomography (PET–CT) showed abnormal FDG uptake from the jejunum to the transverse colon, and the disease was classified as Lugano Stage II. Due to tumor location and the patient's poor general condition, both surgery and chemotherapy were considered infeasible, and best supportive care was chosen. The patient died on hospital Day 33. Jejunocolic fistula caused by MEITL is exceptionally rare. To our knowledge, this is the first reported case in which the fistulous opening was directly visualized endoscopically and a histopathological diagnosis was established by biopsy during life. This case highlights that, in patients with marked weight loss and hypoalbuminemia, gastrointestinal fistula formation should raise suspicion for malignant lymphoma, including MEITL, and that careful endoscopic examination and biopsy may contribute to early diagnosis.

## Graphical abstract

Monomorphic epitheliotropic intestinal T‐cell lymphoma (MEITL) rarely presents with gastrointestinal fistula formation. A rare case of MEITL is identified in an elderly woman with marked weight loss and hypoalbuminemia, in whom a jejunocolic fistula is directly visualized endoscopically. This case highlights the importance of considering malignant lymphoma, including MEITL, in the differential diagnosis of gastrointestinal fistulas in patients with severe malnutrition.

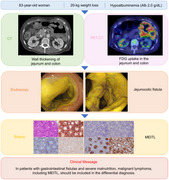

## 1 Introduction

Monomorphic epitheliotropic intestinal T‐cell lymphoma (MEITL) is a primary intestinal T‐cell lymphoma that was previously classified as Type II enteropathy–associated T‐cell lymphoma (EATL Type II) but was redefined as a distinct entity in the 2016 revision of the WHO classification of lymphoid neoplasms [1]. MEITL is exceedingly rare, accounting for fewer than 5% of gastrointestinal lymphomas [2]. Clinically, it often presents with nonspecific symptoms, such as abdominal pain, diarrhea, and weight loss [3], and many patients are already in an advanced stage with perforation, gastrointestinal bleeding, or bowel obstruction at diagnosis [2].

Jejunocolic fistulas can arise secondary to malignant tumors, inflammatory bowel diseases, postoperative complications, ingested foreign bodies, and diverticulitis [4]. However, reports of jejunocolic fistulas caused by gastrointestinal lymphomas are extremely rare, with only a few reported cases associated with diffuse large B‐cell lymphoma (DLBCL) or peripheral T‐cell lymphoma (PTCL).

Here, we describe an unusual case of MEITL complicated by a jejunocolic fistula, in which the fistulous opening was successfully identified endoscopically and a histopathological diagnosis was established by biopsy during life. This case highlights that gastrointestinal fistula formation should be suspected in patients with marked weight loss and hypoalbuminemia. When computed tomography (CT) findings suggest an enteric fistula, malignant lymphoma, including MEITL, should be considered, and careful endoscopic evaluation with appropriate biopsy may contribute to early diagnosis.

## 2 Case Report

**FIGURE 1:**
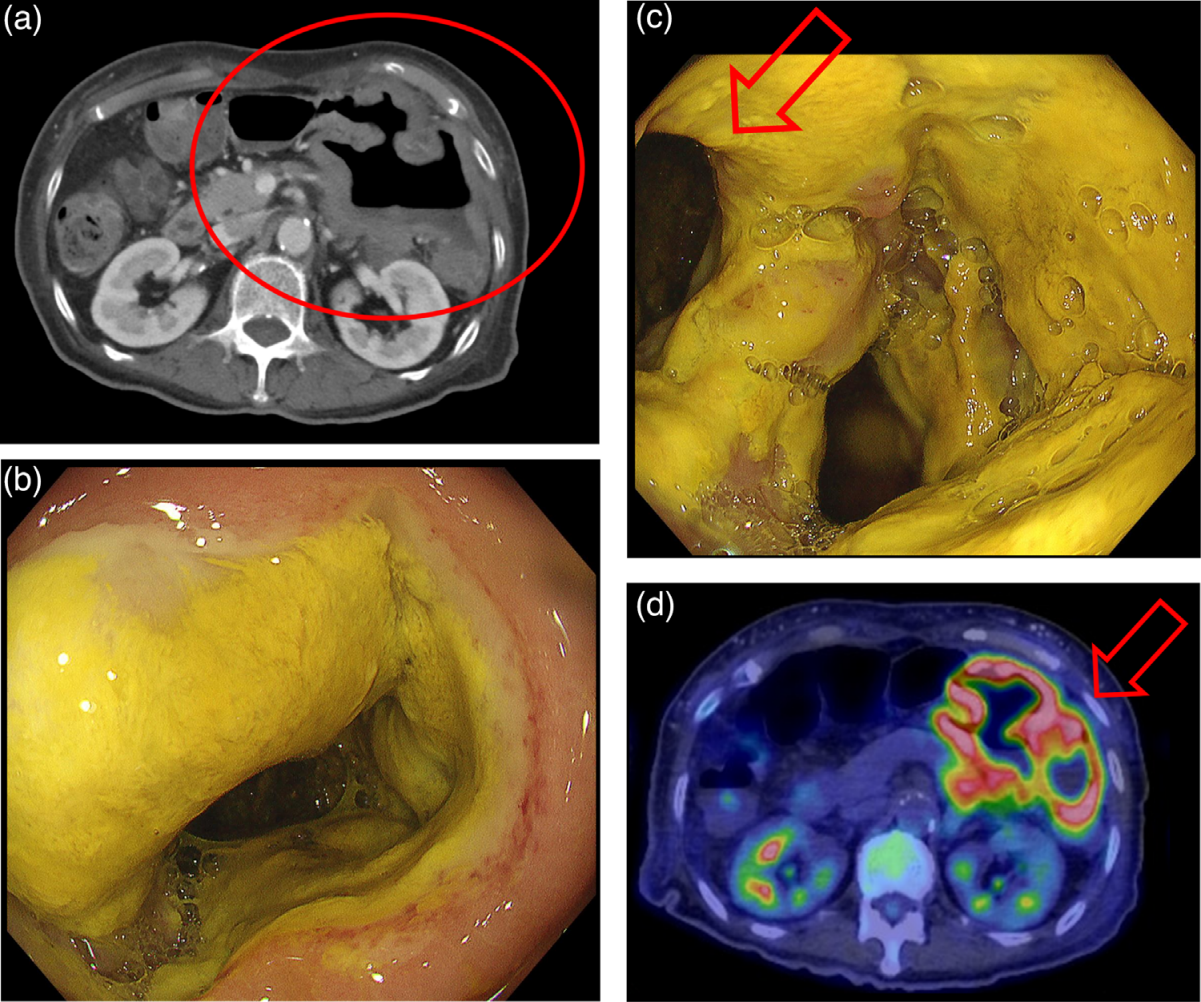
Contrast‐enhanced CT, colonoscopy, and PET–CT findings. (a) Contrast‐enhanced CT demonstrated marked wall thickening and dilatation extending from the splenic flexure to the descending colon. Wall thickening of the proximal jejunum near the ligament of Treitz was also noted, suggesting continuity between the jejunal and colonic lesions (circles). (b and c) Colonoscopy showed a circumferential ulcerative lesion at the splenic flexure. Proximal to the lesion, an additional lumen deviating from the normal colonic lumen was identified (arrows), suggesting the presence of an internal fistula. The lumen at the tumor site was dilated, raising suspicion of aneurysmal‐type malignant lymphoma. (d) PET–CT revealed abnormal FDG uptake in the splenic flexure and descending colon, as well as in the horizontal portion of the duodenum and proximal jejunum (arrows).

**FIGURE 2:**
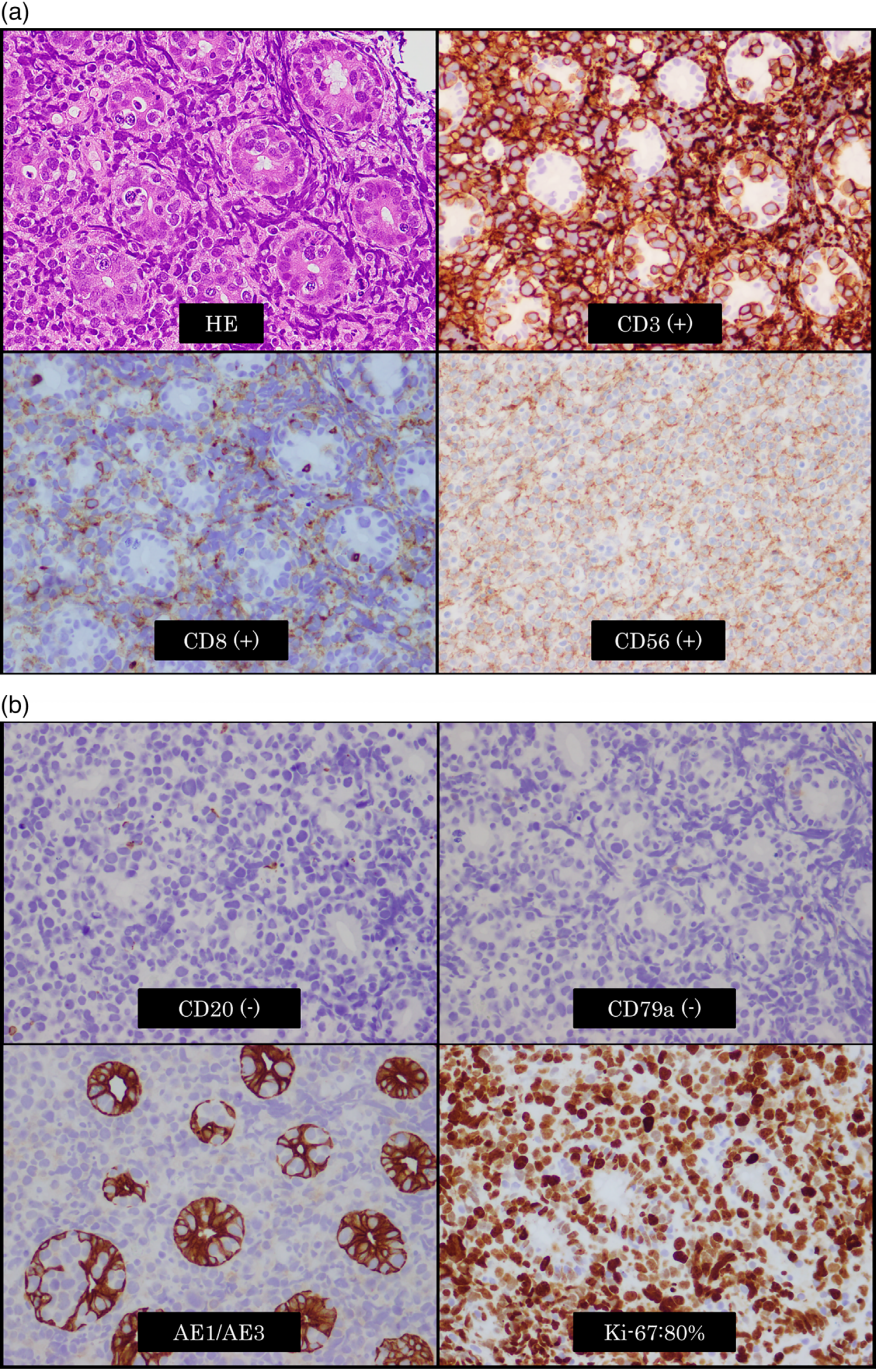
Histopathological findings. (a) Hematoxylin and eosin (H&E) staining and T‐cell markers. Histological examination revealed diffuse proliferation of atypical lymphocytes within the lamina propria, with an epitheliotropic pattern characterized by tumor cells undermining and eroding the epithelium. Immunohistochemical staining demonstrated diffuse positivity of the atypical lymphocytes for CD3, CD8, and CD56. (b) B‐cell, epithelial, and proliferation markers. CD20 and CD79a were negative. Immunostaining for AE1/AE3 highlighted destruction of the glandular structures by the atypical lymphocytes. The Ki‐67 labeling index was elevated at 80%, indicating the highly proliferative nature of the tumor.

An 83‐year‐old woman (height, 150 cm; weight, 41.7 kg; body mass index, 18.5 kg/m^2^) presented to our department for further evaluation after experiencing a 20‐kg weight loss and generalized fatigue over the preceding year. On admission, her body temperature was 37.7°C, and other vital signs were stable. Physical examination revealed a flat and soft abdomen with mild tenderness in the left flank. Her medical history included dyslipidemia and appendectomy. Laboratory tests showed hypoalbuminemia (albumin 2.0 g/dL), elevated C‐reactive protein (CRP 8.0 mg/dL), and an increased soluble interleukin‐2 receptor level (sIL‐2R 1187 U/L) (Table 1). Contrast‐enhanced CT revealed marked wall thickening from the splenic flexure to the descending colon, as well as wall thickening of the proximal jejunum near the ligament of Treitz, suggesting possible continuity between the two segments (Figure 1a). Colonoscopy demonstrated a circumferential ulcerative lesion at the splenic flexure. On the proximal to the lesion, a second lumen was identified that extended in a direction different from the colonic lumen (Figure 1b,c). Because of the risk of perforation, the endoscope was not advanced into the suspected fistula. No procedure‐related adverse events, including abdominal pain or bleeding, were observed after the examination. Positron emission tomography–computed tomography (PET–CT) revealed abnormal fluorodeoxyglucose uptake extending from the splenic flexure to the descending colon, as well as from the horizontal portion of the duodenum to the proximal jejunum, without evidence of lymph node involvement (Figure 1d). Biopsies taken from the ulcerative lesion of the transverse colon demonstrated diffuse proliferation of atypical lymphoid cells with epitheliotropic infiltration and glandular destruction. Immunohistochemical staining showed diffusely positivity for CD3, CD8, and CD56, and negativity for CD20 and CD79a. AE1/AE3 staining highlighted the destruction of glandular epithelium by the infiltrating atypical lymphocytes. The Ki‐67 index was elevated at 80% (Figure 2). These findings were consistent with a diagnosis of MEITL.

**TABLE 1:** Laboratory findings on admission.

Parameter	Result	Reference range
White blood cells (WBC) (×10^3^/µL)	10.1	3.30–8.60
Neutrophils (%)	76.1	40.0–70.0
Lymphocytes (%)	17.1	18.0–53.0
Monocytes (%)	6.2	2.0–12.0
Eosinophils (%)	0.4	1.0–4.0
Basophils (%)	0.2	0.0–1.0
Red blood cells (RBC) (×10^6^/µL)	3.32	3.86–4.92
Hemoglobin (Hb) (g/dL)	10.3	11.6–14.8
Hematocrit (Ht) (%)	31.0	35.1–44.4
Mean corpuscular volume (MCV) (fL)	93.4	83.6–98.2
Platelet count (Plt) (×10^3^/µL)	388	158–348
Total protein (TP) (g/dL)	5.1	6.6–8.1
Albumin (g/dL)	2.0	4.1–5.1
Total bilirubin (mg/dL)	0.5	0.4–1.5
Aspartate aminotransferase (AST) (U/L)	13	13–30
Alanine aminotransferase (ALT) (U/L)	4	7–23
Lactate dehydrogenase (LDH) (U/L)	164	124–222
Alkaline phosphatase (ALP) (U/L)	73	38–113
Amylase (AMY) (U/L)	18	44–132
Total cholesterol (mg/dL)	138	142–248
Blood urea nitrogen (BUN) (mg/dL)	10	8–20
Creatinine (mg/dL)	0.36	0.46–0.79
Sodium (Na) (mmol/L)	140	138–145
Potassium (K) (mmol/L)	2.5	3.6–4.8
Chloride (Cl) (mmol/L)	101	101–108
Calcium (Ca) (mg/dL)	8.1	8.8–10.1
Iron (Fe) (mg/dL)	24	40–188
Ferritin (ng/mL)	339	6.2–138.0
C‐reactive protein (CRP) (mg/dL)	8.0	<0.14
Soluble interleukin‐2 receptor (sIL‐2R) (U/L)	1187	156.6–474.5
Prothrombin time (PT) (s)	12.4	10.0–13.5
Activated partial thromboplastin time (APTT) (s)	24.6	26.0–41.0

We considered the jejunum as the primary site on the basis of the distribution and volume of the tumor on CT, dominant FDG uptake pattern. The disease was staged as Lugano Stage II and Ann Arbor Stage IIB. Because the lesion extended to the region near the ligament of Treitz, surgical resection was deemed excessively invasive and not feasible given the patient's overall condition. Chemotherapy was also considered high risk due to the potential for perforation and poor performance status. After discussion with the patient and her family, a best supportive care (BSC) approach was selected. The patient died on hospital Day 33.

## 3 Discussion

In 1946, De Nadal first reported a gastrointestinal fistula caused by lymphoma, and since then, various types of fistulas—including gastrosplenic, choledochoduodenal, and duodenocolic fistulas—have been described in association with gastrointestinal lymphomas. In our literature review, only two cases of jejunocolic fistulas caused by T‐cell lymphomas were identified, and none were diagnosed as MEITL or EATL Type II. To the best of our knowledge, the present case represents the first reported instance of MEITL complicated by a jejunocolic fistula.

A summary of previously reported cases of jejunocolic fistulas caused by gastrointestinal lymphoma is provided in Table 2. A total of five cases have been reported: three involving DLBCL, and one each involving PTCL and diffuse aggressive T‐cell lymphoma. All five cases presented with diarrhea and weight loss, whereas some also had fever or abdominal pain. CT and endoscopy were useful for diagnosis in most cases, although biopsy specimens were occasionally nondiagnostic and surgical specimens were required for definitive diagnosis. Prognosis was generally poor, particularly in T‐cell lymphoma complicated by perforation.

**TABLE 2:** Summary of previously reported cases of jejunocolic fistulas caused by gastrointestinal lymphoma.

Year	Author	Age/Sex	Past Medical History	Symptoms	Imaging Studies	Diagnostic Specimen	Histopathological Findings	Clinical Course
2005	L.E. McMahon	61/F	Status post cholecystectomy	Diarrhea, weight loss	CT scan, endoscopy	Surgical specimen (biopsy non‐diagnostic)	Diffuse aggressive T‐cell lymphoma	Postoperative CHOP chemotherapy; alive without recurrence at 10 months after surgery
2007	V. Wang	86/F	Chronic obstructive pulmonary disease	Abdominal pain, diarrhea, weight loss	CT scan, endoscopy	Biopsy specimen	DLBCL	Best supportive care
2009	A. Jangjoo	36/M	None	Diarrhea, abdominal pain, weight loss	Upper gastrointestinal contrast study	Surgical specimen	DLBCL	Postoperative chemotherapy; further clinical course unknown
2011	H.B. Chun	56/M	Intestinal tuberculosis, chronic hepatitis B	Intermittent fever, diarrhea, weight loss	CT scan, endoscopy	Surgical specimen (biopsy nondiagnostic)	PTCL	Died of peritonitis and sepsis 2 months after surgery
2022	R.N. Jr. Velasco	20s/M	Superior vena cava syndrome	Diarrhea, bleeding, weight loss	CT scan, endoscopy	Surgical specimen	DLBCL	Postoperative chemotherapy; died 33 months after symptom onset

Abbreviations: CT, computed tomography; DLBCL, diffuse large B‐cell lymphoma; PTCL, peripheral T‐cell lymphoma.

The most notable feature of this case is that the jejunocolic fistula was directly visualized endoscopically, and a definitive diagnosis of MEITL was established solely on the basis of endoscopic biopsy specimens.

Fistula formation secondary to gastrointestinal malignant lymphoma is rare, and its underlying pathogenesis remains poorly understood. Malignant lymphomas are believed to predispose to perforation because the tumor cells infiltrate the entire bowel wall with minimal fibrocollagenous reaction, rendering the bowel structurally fragile compared with carcinoma [5]. In addition, T‐cell lymphomas show angiocentric infiltration, causing tissue liquefaction and necrosis, which further increases the likelihood of perforation [6].

MEITL is also thought to exhibit similarly destructive patterns of infiltration, which can lead to bowel perforation and fistula formation [5]. Fistula development is considered a progressive process resulting from transmural tumor infiltration and adhesion to adjacent bowel loops [7]. In the present case, ulcer formation likely increased the risk of penetration. Furthermore, the high mobility of the jejunum and its anatomical proximity to the transverse and descending colon may have facilitated the formation of the fistula.

Jejunocolic fistulas are often difficult to diagnose due to nonspecific symptoms. Abdominal pain, diarrhea, and significant weight loss are among the most commonly reported [8], and all previously reported cases presented with both diarrhea and weight loss. These symptoms likely result from the diversion of small‐intestinal contents directly into the colon, causing malabsorption similar to short bowel syndrome [9]. In the present case, the patient exhibited severe weight loss and hypoalbuminemia, findings that likely reflect not only the systemic impact of MEITL but also the pathophysiological consequences of the jejunocolic fistula.

CT is the first‐line modality for diagnosing tumor‐associated enteric fistulas. Intratumoral gas or visualization of a tract between the tumor and bowel strongly suggests internal fistula formation [10].

Histopathological confirmation remains essential for diagnosing malignant intestinal lesions, and endoscopic evaluation plays a key role by enabling direct visualization and tissue sampling. However, endoscopic procedures carry risks of bleeding and perforation, and deep small intestinal lesions may not always be reachable. As reported in previous cases, superficial mucosal biopsies may be nondiagnostic or falsely negative, with some cases requiring surgical specimens to establish a definitive diagnosis. In this case, we were able to reach the fistulous opening endoscopically and obtain diagnostic tissue, making this a particularly valuable clinical experience.

Although surgical resection and chemotherapy were considered, both approaches were deemed high risk due to tumor location and the patient's overall condition, and BSC was ultimately selected. Given the extremely poor prognosis of MEITL, early diagnosis and timely intervention are important.

We report a rare case of MEITL presenting with a jejunocolic fistula that was successfully identified endoscopically. This case highlights the importance of the pathogenesis of lymphoma‐associated fistulas, underscoring the significance of early recognition and diagnosis.

## Author Contributions

Kanami Ota managed the patient, collected the clinical data, and drafted the manuscript. Yuichi Matsuno managed the patient, performed the endoscopic examinations, and edited the manuscript. Yoshiaki Taniguchi performed the pathological evaluation and interpreted the histological findings. Noriyuki Imazu, Shinichiro Kawatoko, and Yutaro Ihara provided diagnostic advice. Tomohiro Nagasue, Keisuke Kawasaki, and Tomohiko Moriyama evaluated and interpreted the imaging findings. Junji Umeno supervised the case and critically reviewed the manuscript for important intellectual content. All authors reviewed and approved the final version of the manuscript.

## Funding

The authors have nothing to report.

## Conflicts of Interest

The authors declare no conflicts of interest.

## Data Availability

The data that support the findings of this study are available from the corresponding author upon reasonable request.
